# Aerobic Dance on an Air Dissipation Platform Improves Cardiorespiratory, Muscular and Cellular Fitness in the Overweight and Obese Elderly

**DOI:** 10.3390/biology11040579

**Published:** 2022-04-11

**Authors:** Alessandra Moreira-Reis, José Luis Maté-Muñoz, Juan Hernández-Lougedo, Sergio Vilches-Sáez, Marta Benet, Pablo García-Fernández, Eulogio Pleguezuelos, Teresa Carbonell, Norma Alva, Manuel Vicente Garnacho-Castaño

**Affiliations:** 1 Department of Cell Biology, Physiology and Immunology, Faculty of Biology, University of Barcelona, 08028 Barcelona, Spain; alereis55@hotmail.com (A.M.-R.); svilches@escs.tecnocampus.cat (S.V.-S.); tcarbonell@ub.edu (T.C.); nvalva@ub.edu (N.A.); 2 Department of Radiology, Rehabilitation and Physiotherapy, Complutense University of Madrid, 28040 Madrid, Spain; jmate03@ucm.es (J.L.M.-M.); pablga25@ucm.es (P.G.-F.); 3 Laboratory of Biomechanics and Exercise Physiology, Department of Physical Activity and Sports Science, Alfonso X El Sabio University, 28691 Madrid, Spain; jhernlou@uax.es; 4 Campus Docent Sant Joan de Déu, University of Barcelona, 08034 Barcelona, Spain; marta.benet@sjd.edu.es; 5 IdISSC, Instituto de Investigación Sanitaria del Hospital Clínico San Carlos, 28040 Madrid, Spain; 6 Physical Medicine and Rehabilitation Department, Hospital de Mataró, 08304 Barcelona, Spain; epleguezuelos@csdm.cat

**Keywords:** obesity, body composition, elderly, oxidative stress, human performance, cardiorespiratory fitness, aerobic dance, health promotion

## Abstract

**Simple Summary:**

Aerobic dance is considered a viable strategy to prevent the effects of aging, mainly in obese and overweight elderly people. This study aimed to evaluate the effects of aerobic dance on an air dissipation platform (ADP) on body composition, oxidative stress and muscular and cardiorespiratory fitness in 32 elderly adults (67.1 ± 3.6) who were divided into 3 groups based on body mass index: healthy (HG), overweight (OWG) and obese (OG). Training program of aerobic dance on an ADP was carried out twice a week for 12 weeks at an intensity of 6–8 on the scale of subjective perception exertion (Borg Scale, CR-10). There was a significant decrease in malondialdehyde concentrations in all experimental groups. OWG and OG significantly improved their peak oxygen uptake. An interaction effect was observed in vertical flight height and power output, during the jump test. HG increased the vertical jump height, and HG and OG improved the power output of the lower extremities. In conclusion, aerobic dance on an ADP may be an effective alternative to lose weight, prevent oxidative stress and improve cardiorespiratory fitness in obese and overweight elderly people.

**Abstract:**

Background: Obesity is a global health problem associated with a high number of comorbidities that decrease functional capacity, especially in elderly people. Aerobic dance is considered a viable strategy to prevent the effects of aging, mainly in obese and overweight elderly people. This study aimed to evaluate the effects of aerobic dance on an air dissipation platform (ADP) on body composition, oxidative stress and muscular and cardiorespiratory fitness in elderly people. Methods: In total, 32 elderly adults (67.1 ± 3.6) were divided into 3 groups based on body mass index: healthy (HG), overweight (OWG) and obese (OG). Training program of aerobic dance on an ADP was carried out twice a week for 12 weeks. Results: OWG (*p* = 0.016) and OG decreased their weight (*p* < 0.001). There was a significant decrease in malondialdehyde concentrations in all experimental groups (*p* < 0.05). OWG and OG significantly improved their peak oxygen uptake (*p* < 0.01). HG increased the vertical jump height (*p* < 0.05), and HG and OG improved the power output of the lower extremities (*p* < 0.05). Conclusions: The aerobic dance on an ADP may be an effective alternative to lose weight, prevent oxidative stress and improve cardiorespiratory fitness in obese and overweight elderly people.

## 1. Introduction

Obesity is a global health problem associated with a high number of comorbidities that affect quality of life and decrease functional capacity, especially in elderly people. Globally, the prevalence of obesity in adults increased from 6.4% to 14.9% in women and from 3.2% to 10.8% in men between 1975 and 2014 [1]. Population studies have shown the relationship between a body mass index (BMI) higher than 25 kg/m^2^ (especially a BMI ≥ 30 kg/m^2^) and greater functional impairment [2,3]. In elderly people, BMI ≥ 25 kg/m^2^ is related to chronic diseases, metabolic syndrome, diabetes [4], frailty [5] and increased mortality [5,6].

At the cellular level, increased concentrations of reactive oxygen and nitrogen species (RONS), combined with the reduction in endogenous antioxidants are common features for both, the aging process and obesity, increasing oxidative stress [7,8,9]. The imbalance between the antioxidant systems and free radical overproduction leads to cell oxidative damage affecting tissue components such as lipids, proteins or deoxyribonucleic acid (DNA) molecules [10]. 

Obesity leads to an increase in adipose tissue, triggering the release and storage of lipids in the skeletal muscle. These intramuscular lipids and their derivatives induce a mitochondrial dysfunction characterized by alterations in β-oxidation capacity, therefore, increasing oxidative stress (ROS) and impairing metabolic function [11]. The main products of lipid peroxidation are lipid hydroperoxides where malondialdehyde (MDA) is commonly formed as a secondary by product. It has been described that lipid peroxidation is greater in skeletal muscle mass in obese adults [12]. Several studies have shown that sarcopenia, high BMI and increased MDA concentrations are all parameters [13,14,15] related to an augmented risk of cardiovascular diseases [16,17] and incidence of atherosclerotic processes involving circulating lipoproteins [10]. 

Countless studies point to regular physical activity as one of the most beneficial resources for delaying the physiological deterioration induced by aging and obesity [18,19,20]. Specifically, aerobic dance (AD) is one of the most practiced aerobic activities in the world, mainly in senior centers [21]. AD is characterized by a sequence of impact movements choreographed to the rhythm of the music. Several studies have proposed that AD improves muscular strength, cardiorespiratory endurance, body agility, flexibility [22,23], lower body function [24] and locomotion/agility and balance, thus attenuating risks of falling in elderly adults [25]. AD exercise programs have been shown to reduce body weight, fat mass and cardiovascular disease risks in overweight and obese women [26], as well as improve maximal oxygen uptake (VO_2_), decrease MDA levels and enhance antioxidant capacity [27]. From a psychological perspective, AD has been confirmed to have a positive effect on cognition in older people [28].

Exercise programs for the elderly that include unstable surfaces have been proposed to induce improvements in physical capabilities, such as muscle strength, power and balance [29,30], functional mobility, gait performance [19] and appear to be a good alternative to reduce the impact on joints [31]. Unstable surfaces have been shown to be a suitable alternative for improving cardiorespiratory fitness and producing positive changes in body composition in overweight women [32]. In addition, several studies have shown that exercise performed on unstable surfaces can be more intense compared to exercise on the ground [33,34,35]. 

Recently, our research group incorporated an air dissipation platform (ADP) in AD sessions. The ADP contains an area that rests on an elastomer with holes through which air flows. The amount of air that remains in the area produces rebound damping, reducing impacts during exercise and increasing instability. In a previous study, we demonstrated that an AD session on an ADP increased metabolic and cardiorespiratory responses compared to a hard surface, maintaining the perception of greater effort and muscle fatigue. [35]. We suggested that an AD exercise program on an ADP carried out 3 d·wk^−1^ for 75 min·wk^−1^ or 20 min·d^−1^ could maintain or improve metabolic and cardiorespiratory fitness, according to the American College of Sports Medicine (ACSM) guidelines [18].

To our knowledge, there are no studies assessing the effects of AD on an ADP on oxidative stress and cardiorespiratory and muscular function in obese or overweight older people. Therefore, this study aimed to investigate the chronic effects of an exercise program of AD performed in an ADP on cardiorespiratory and muscular fitness and oxidative stress in overweight and obese older people.

## 2. Materials and Methods

### 2.1. Subsection

The exercise program was explained in detail to the participants in the preliminary meeting. In the first session, all subjects were rigorously evaluated for comorbidities and diseases and their medical history was analyzed. In addition, their level of physical activity was checked up using an international physical activity questionnaire (IPAQ-E) for measurement of physical activity in people over 65 years of age [36].

**Figure 1:**
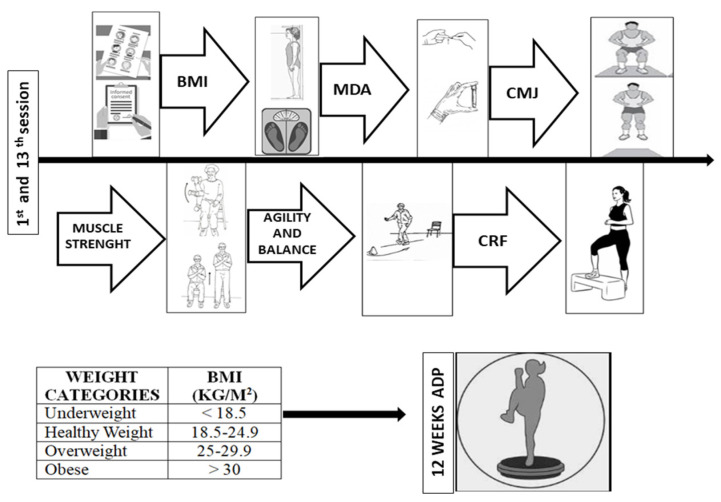
Protocol test. Abbreviations: BMI = body mass index, CMJ = counter movement jump, CRF = cardiorespiratory fitness and MDA = malondialdehyde. The images were selected from the internet (20 September 2021). http://fonamentsgrausuperiordanigoncalves.blogspot.com/p/senior-fitness-test.html; https://www.klipartz.com/es/sticker-png-ouoxs; https://www.makeoverfitness.com/leg-exercise-charts/7906-printable-leg-exercise-chart-for-women; https://mundoentrenamiento.com/salto-vertical-como-aumentarlo/; https://www.pngwing.com; https://www.shutterstock.com/es/search/chemistry+sketches; https://sp.depositphotos.com/vector-images/consentimiento-informado.html.

The AD program in an ADP lasted 12 weeks. Before (pre-test) and after (post-test) the AD program, the same tests were carried out by the same evaluators to determine the effects of the exercise program in an ADP on body composition, oxidative stress and cardiorespiratory and muscular fitness. Previously, a familiarization session was performed of the muscular and cardiorespiratory fitness tests. The participants did not perform any physical effort for 48 h before the tests. The tests and the order of the tests were defined as follows (Figure 1): 1st assessment of body composition, 2nd capillary blood collection, 3rd assessment of muscular fitness (lower extremity strength, upper extremity strength, jump test), 4th assessment of agility and dynamic balance (8 foot UP & Go test) and 5th assessment of cardiorespiratory fitness (YMCA test). A 5 min rest was established between each test.

### 2.2. Participants

Participants of this study were members of the centers for the elderly in the community of Madrid. In total, 58 healthy older adults between 60 and 78 years old were recruited. Finally, 32 participants (age = 67.1 ± 3.6 years; weight = 67.5 ± 16.6 kg; height = 155.4 ± 6.7 cm; BMI = 27.9 ± 6.2 kg·m^2–1^) were included in this study. In total, 28 women and 4 men were assigned to 3 groups based on BMI according to established guidelines by the World Health Organization [37]: eutrophic (18.5–24.9 kg m^2^), overweight (25–29.9 kg/m^2^) and obese (≥30 kg/m^2^): healthy group (HG, *n* = 10; men, *n* = 1), overweight group (OWG, *n* = 10; men, *n* = 2) and obese group (OG, *n* = 12; men, *n* = 1).

Participants with orthopedic prostheses or implanted pacemaker, cardiovascular neurological, musculoskeletal, infectious and oncological diseases were excluded from the study. In addition, all participants who missed 10% of the exercise sessions in the ADP were excluded from the final data analysis. The participants were informed of all experimental procedures and each participant provided written informed consent to participate in the study. This investigation was approved by the Institutional Review Board (Identification number: 13/2018) according to the principles and policies of the Declaration of Helsinki.

### 2.3. Body Composition

Body composition was calculated by bioimpedance using the electric Bioimpedance model scale (InBody 3.0, Biospace, Seoul, Korea). The variables assessed were weight, body fat (BF), body fat percentage (% BF), fat-free mass (FFM) and lean mass (LM) [38]. Height was measured with a standard stadiometer, and BMI was calculated as weight (kg)/height (m^2^).

### 2.4. Oxidative Stress

Blood samples for oxidative stress determination were collected by finger pricking. After puncture, blood was immediately collected in EDTA-K2 Microvette tubes (SAR-STEDT, Nümbrecht, Germany). The tubes were centrifuged at 600× *g* for 15 min (4 °C). To avoid peroxidation amplification, butylated hydroxytoluene (antioxidant) and the iron chelator EDTA were added to fresh plasma samples. Then, plasma was stored at −80 °C until assessment of MDA as determined by thiobarbituric acid-reactive substance concentrations (TBARS), a product of lipid peroxidation, following Yagi’s technique with minor modification [39]. Results were expressed in uM compared to a standard curve prepared with MDA.

### 2.5. Muscular Fitness

After a 5 min general warm-up (movements, joint mobility, push-ups, jumps, etc.), the participants began the assessment of muscle fitness.

#### 2.5.1. Countermovement Jump

The countermovement jump (CMJ) was used to measure the vertical flight height and the power output of the lower extremities on a contact platform (ChronoJump, Bosco System, Barcelona, Spain). Three CMJs were performed at the participant’s maximum capacity with a 30 s rest between each jump. The mean values of height and mean power output of the three jumps were used in the subsequent analyses [40].

#### 2.5.2. Arm Curl Test

Sitting, at the signal of the evaluator, the participant performed an elbow flexion–extension (bicep flexion) with both limbs throughout the range of motion as many times as possible for 30 s. The test started with the dominant arm and ended with the non-dominant. A single series was performed and 1 min rest was established between each attempt. A 2 kg dumbbell was used for women and 4 kg for men [41].

#### 2.5.3. Agility and Dynamic Balance

Agility and dynamic balance were assessed using the 8 foot UP & Go test. At the investigator’s signal, the participant should get up from the chair and walk 8 feet (2.44 m), turn around and sit back down. Two attempts were executed and the shortest time of the two attempts was recorded [42]. A 3 min rest was applied between attempts.

### 2.6. Cardiorespiratory Fitness

The cardiorespiratory fitness was assessed using the YMCA step test. At a 30 cm high stride, participants performed 24 steps per minute at a rate of 96 bits per minute for 3 min [43]. Heart rate values were recorded using a polar heart rate monitor (RS-800CX, Polar Electro OY; Kempele, Finland) during the exercise and 1 min after exercise (1 min HBC). Peak oxygen consumption was estimated according to the guidelines established in a previous study by Beutner et al., who established a linear regression model (YMCA model) taking into account age, sex and 1 min HBC. The regression coefficients for each of the variables were: −0.15 for 1 min HBC, −4.2 for the gender variable, −0.38 for the age variable and 78.2 as a constant [44].

### 2.7. Exercise Program

**Figure A1:**
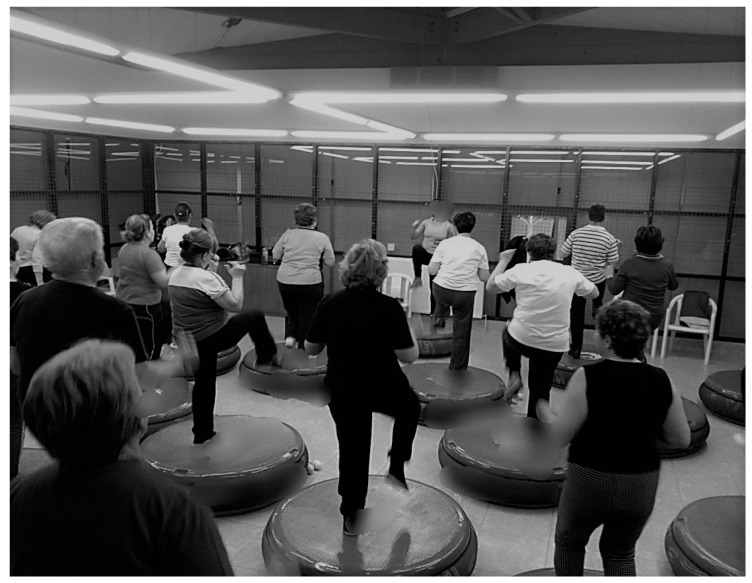
Session of ADP with the elderly.

All sessions were led by the same instructor. Two AD classes on an ADP were conducted per week for 12 weeks (Appendix A, Figure A1). The duration of the classes was 45 min divided into 10 min of warm-up, 30 min of the main part and 5 min of cool down. The AD class consisted of global and combined lower and upper body exercises such as jumps with both feet, knee raises, flexion with elbow extension, kick with shoulder abduction, squats, leg flexion and extension, jumping jacks, scissors, calisthenics, plyometrics etc. The music that accompanied the exercises was selected to mark the right time of transition between the different types of exercise. The exercise changes were performed every 16 s and the intensity of the class was controlled by the Borg rating of perceived exertion (RPE, Borg Scale CR-10) [45] following the guidelines established in previous studies [46]. Upper body exercises consisted of performing elbow flexion–extensions, shoulder abductions–adductions and shoulder flexion–extension with dumbbells and elastic bands simultaneously while dancing to the rhythm of the music. The participants had to perform a high number of repetitions (≈15 repetitions) with light resistance. After 16 s, the muscle group was changed to another upper extremity exercise (Table 1).

**Figure A2:**
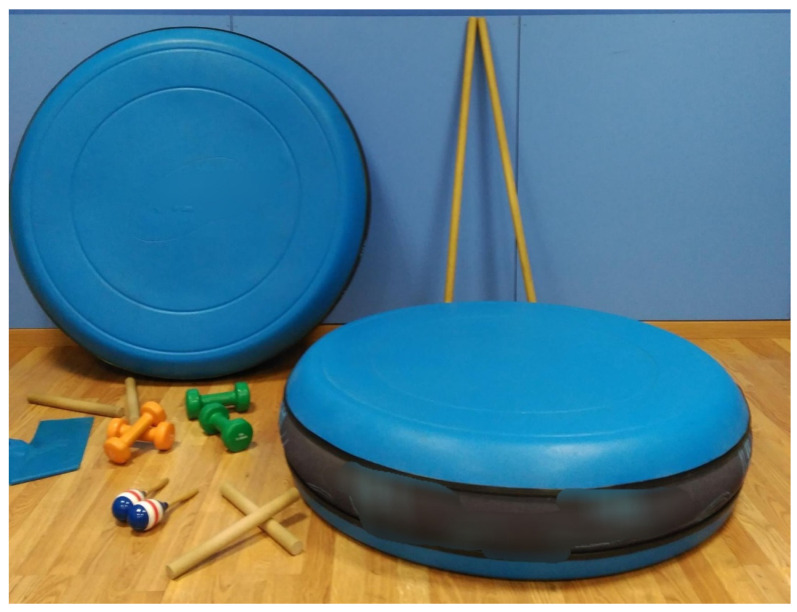
The air dissipation platform (ADP), materials such as dumbbells, elastic bands, maracas, sticks and paddles used in the sessions.

In addition to performing all the exercises on the platform, materials such as dumbbells, rubber bands, maracas, sticks and pikes were also used (Appendix A, Figure A2).

### 2.8. Statistical Analysis

The Shapiro–Wilk test was used to check the normal distribution of the data, which are reported as means and standard deviation (SD), means and confidence intervals (95% CI). To identify significant differences between the HG, OWG and OG, a general linear model with a two-way analysis of variance (ANOVA) for repeated measures was applied (group × time). When appropriate, a post hoc Bonferroni adjustment was implemented for multiple comparisons. The partial eta-squared (η_p_^2^) was computed to determine the magnitude of the response to exercise program. The statistical power (SP) was also calculated. All statistical tests were performed using the software package SPSS version 23.0 for Apple Macintosh (SPSS Inc., Chicago, IL, USA). Significance was set at *p* < 0.05.

## 3. Results

### 3.1. Body Composition

The data related to body composition are shown in Table 2.

### 3.2. Oxidative Stress

**Figure 2:**
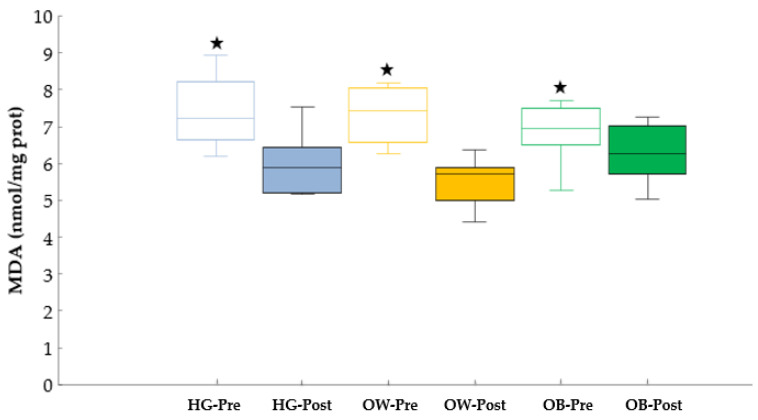
Lipid peroxidation, measured as TBARS concentrations. Abbreviations used: HG = healthy group; MDA = malondialdehyde; OG = obesity group; OWG = overweight group. ★ Significant differences compared to posttest (*p* < 0.001 in HG; *p* < 0.001 in OWG; *p* = 0.024 in OG).

In MDA, an interaction effect (group × time) and a time effect (*p* = 0.032, ES = 0.25, SP = 0.66; *p* < 0.001, ES = 0.70, SP = 1.00, respectively) were verified; however, a group effect was not detected (*p* > 0.05). The Bonferroni post hoc determined a significant decrease in MDA concentrations in the three experimental groups after the training program (*p* < 0.05) (Figure 2).

### 3.3. Cardiorespiratory Fitness

The data related to cardiorespiratory and muscular fitness are shown in Table 3.

Regarding the estimated VO_2peak_, an interaction effect (group × time) (*p* = 0.008) was observed. The Bonferroni test determined that the OWG and OG significantly improved their VO_2peak_ (*p* = 0.005 and *p* = 0.002, respectively). No interaction effect (group × time) was detected in the strength of the arms and in the 8 foot UP & Go test (*p* > 0.05). However, an interaction effect (group × time) was observed in vertical flight height (*p* = 0.001) and power output (*p* = 0.044) during the jump test. Bonferroni test determined that only HG increased the vertical jump height after training program (*p* < 0.05). Furthermore, the power output of lower limbs was improved in HG and OG after the training program (*p* < 0.05).

## 4. Discussion

The main finding of this study was that a 12-week AD exercise program on an ADP successfully reduced body weight, decreased lipid peroxidation (MDA) and increased VO_2_peak in obese and overweight elderly. Moreover, the OG showed an improvement in balance and agility and also in the strength of both arms over time (pre vs. post). One of the main objectives of an exercise program in obese and overweight people is to lose weight and gain lean mass. Our results indicated that only OWG and OG decreased their weight after the intervention program while HG maintained weight and lean mass. It should be noted that weight loss in elderly may have an effect on reducing lean mass, which could increase the risk of sarcopenia [3,47]. However, no significant changes in lean mass in OWG and OG were detected after the AD program; although aerobic exercise compared to other types of exercise has less effect on lean mass [47,48]. It has been evidenced that weight loss and maintenance of lean mass decrease the risk of developing metabolic diseases, reducing skeletal muscle deterioration and disability, hospitalizations and early mortality [49]. An exercise program of two sessions per week of AD in an ADP for at least 12 weeks could be a sufficient stimulus to reduce weight and maintain lean mass, reducing the risk of metabolic diseases and the deterioration of muscle mass in obese and overweight older people. More studies are needed to corroborate such claims. The role of oxidative stress in the aging process appears mainly related to the decrease in antioxidant systems, and the loss of functionality of other detoxifying systems, causing the accumulation of oxidized lipids, proteins or DNA molecules, negatively impacts on the homeostatic cellular mechanisms [7,8,9].

The level of lipid peroxidation was similar in all the experimental groups before starting the exercise program. At the end of the AD program, the MDA levels were significantly attenuated. Although MDA levels have not been investigated after an exercise program on an ADP, the results were similar to several studies in which other exercise programs were applied [27,50]. Yu et al. showed that aerobic exercise such as running, cycling and dancing induced lower MDA levels and protective effects against oxidative stress damage in older people [50]. Similar findings were found in obese elderly women after performing an aerobic exercise program for 12 months at an intensity of 60–75% of maximum HR. The authors concluded that aerobic exercise decreases oxidative stress when accompanied by gains in cardiorespiratory fitness [51]. From a physiological perspective, the decrease in oxidative stress may be related to an improvement in mitochondrial function. Mitochondrial dysfunction is one of the characteristics of the aging process inducing an elevated emission of ROS and the activation of apoptotic pathways [52]. It seems that exercise programs could reduce oxidative stress in the elderly and obese people, depending on the type of exercise and the intensity established [53,54]. Previous studies found different levels of lipid peroxidation and TBARS in obese individuals at several types of exercise and intensities [55,56,57].

One of the purposes of this study was to be able to control exercise intensity on an ADP. Our previous findings demonstrated that exercise on an ADP stimulates a greater cardiorespiratory and metabolic response compared to exercise on a hard surface [35]. The exercise intensity of the sessions was controlled using the Borg scale of 0 to 10. The instructor regularly reported the ranges and intensity changes, following the aforementioned scale, which allowed the control of intensity levels within the ranges of moderate to vigorous intensity (RPE 5 to 8), ensuring that the subjects were not exposed to strenuous efforts [46]. Studies have shown to improve the cardiorespiratory fitness by controlling exercise intensity with RPE [46,58]. OWG and OG increased their VO_2peak_ at the prescribed intensities, demonstrating the efficacy of the exercise program on an ADP. In contrast, HG did not improve their VO_2peak_, suggesting that the implemented exercise program (2 sessions per week) could be sufficient stimulus to improve VO_2peak_ in overweight and obese older people, but not for older people with a normal weight. Improvements in VO_2peak_ are of crucial relevance to the health of obese and overweight people. Several studies have shown that an increase of 1 MET (3.5 mL/kg/min O_2_) in exercise capacity reduced the adjusted risk for mortality in 13% [59] and reduces the risk of mortality or suffering a cardiovascular event by 13–15% [60]. OWG and OG increased ~1.5 mL/kg/min O_2_ similar to other AD studies using a mini trampoline [27,61]. Cugusi et al. found a significant increase in VO_2max_ (1.5 mL/kg/min, from 15.4 to 16.9 mL/kg/min,) in overweight women after a 12-week exercise program on a mini trampoline [32].

Van Schoor et al. assumed that the higher physiological demands induced by the mini trampoline could be due to the constant rebounding and instability produced by an elastic surface [61]. This higher physiological demand would imply a greater effort to perform the exercise and maintain balance on an ADP. In addition, soft surfaces can reduce the risk of high-impact joint injuries by improving balance and strength in older people [29,30], especially in obese and overweight people. This suggestion could account for, at least in part, the improvements in agility and balance observed in obese older people. However, the exercise program on an ADP was not adequate stimulus to significantly improve agility and balance in the OWG and HG. Nonetheless, the results of the 8 foot UP & Go test can be considered normal [62]. Contrary to what might be expected, the rebound effect produced by ADP did not improve jumping ability in overweight and obese people despite including plyometric in the AD program. However, HG improved vertical jump ability by demonstrating increased lower extremity strength, consistent with other studies where jump training appears to be more effective in non-obese older people [63].

A twice-weekly AD program on an ADP preserved explosive strength levels in the lower extremities in overweight and obese older people. Preventing loss of muscle strength, as well as cardiorespiratory fitness, is crucial for the elderly to maintain their functional ability to perform activities of daily living independently [59,64,65]. Muscle mass declines by roughly 3–8% per decade after the age of 30 and increases even more after the age of 60 [66,67]; this gradual decrease in muscle mass is accompanied by a simultaneous reduction in strength [68], in muscle performance and a decrease in cardiorespiratory fitness [59,64,69,70]. In addition, as a preventive measure, this type of training with instabilities could improve neuro-muscular and musculoskeletal functions and reduce the risk of falls, using exercises for strength, postural balance, muscle coordination, joint range of motion and spatial orientation with a multi-component approach [29,30].

This study presents some limitations. Some participants did not attend the posttest, which significantly reduced the sample size. The initial sample of 58 participants was reduced to 32 participants at the end of study. Data from participants who failed to complete more than two AD sessions were not considered for the final statistical analysis [46] but they continued to perform the exercise program.

## 5. Conclusions

In conclusion, a training program of aerobic exercise on an ADP should be considered a viable strategy to positively regulate cardiorespiratory and muscular adaptations and to ameliorate the effects of oxidative stress in obese and overweight older people.

## Figures and Tables

**Table 1:** Summary of characteristics and expected RPE of dance session performed on an air dissipation platform.

Variable/Weeks	1–3	4–8	9–12
Sessions for week	2	2	2
Exercise intensity	moderate	intensity	vigorous
Expected RPE (1–10)	5–6	6–7	7–8

**Table 2:** Body composition variable.

	Assessment	HG	OWG	OG	P1 ^for interaction^/ES/SP	P2 ^for time/^ES/SP	P3 ^for group^/ES/SP
Participants (*n*)		10	10	12			
Weight (kg)	Pre ^†^	58.20 (4.21)	68.29 (5.70)	80.16 (9.94)	0.028	0.001	<0.001
	Post ^†^	58.22 (3.57)	67.51 (5.61) *	78.98 (9.54) *	0.23/0.68	0.33/0.95	0.62/1.00
Body mass index (kg·m^−2^)	Pre ^†^	24.23 (1.24)	28.83 (1.13)	32.17 (1.93)	0.121	0.002	<0.001
	Post ^†^	24.16 (0.99)	28.59 (0.87)	31.71 (1.95) *	0.14/0.42	0.30/0.92	0.84/1.00
Fat Mass (kg)	Pre ^¥^	22.02 (3.28)	26.22 (2.51)	31.65 (4.54)	0.732	0.078	<0.001
	Post ^¥^	21.72 (2.69)	25.46 (2.17)	31.30 (4.22)	0.02/0.09	0.11/0.42	0.61/1.00
Body Fat (%)	Pre	36.79 (4.29)	38.56 (3.43)	40.78 (6.32)	0.901	0.038	0.180
	Post	36.23 (3.78)	37.74 (3.56)	40.25 (5.98)	0.01/0.06	0.15/0.56	0.12/0.35
Fat-Free Mass (kg)	Pre	37.91 (3.91) ^β^	41.78 (5.09)	46.72 (9.99)	0.971	0.095	0.030
	Post	38.20 (3.83) ^β^	42.15 (5.31)	47.13 (9.76)	0.00/0.05	0.10/0.39	0.23/0.67
Lean Mass (kg)	Pre	20.91 (2.70) ^β^	22.92 (3.01)	25.81 (6.16)	0.401	0.960	0.041
	Post	20.57 (2.33) ^β^	23.02 (3.19)	26.02 (6.08)	0.07/0.20	0.00/0.05	0.21/0.62
Basal Metabolic Rate (kcal·day^−1^)	Pre	1182.11 (82.28) ^β^	1276.20 (108.51)	1383.64 (224.23)	0.775	0.155	0.027
	Post	1195.89 (82.62) ^β^	1281.20 (114.61)	1389.00 (211.44)	0.02/0.09	0.07/0.292	0.24/0.68

Data are provided as mean ± standard deviation (SD). Abbreviations: ES = effect size; HG = healthy group; OG = obesity group; OWG = overweight group; SP = statistical power. P1 = *p*-value for group × time interaction effect; P2 = *p*-value for time effect; P3 = *p*-value for group effect. Bonferroni’s multiple comparisons determined: * Significant differences compared to pretest (*p* < 0.05). ^†^ Significant differences between groups in pretest (HG vs. OWG, *p* ≤ 0.018; HG vs. OG, *p* < 0.001; OWG vs. OG, *p* ≤ 0.002) and posttest (HG vs. OWG, *p* ≤ 0.023; HG vs. OG, *p* < 0.001; OWG vs. OG, *p* ≤ 0.002). ^¥^ Significant differences between groups in pretest (HG vs. OG, *p* < 0.001; OWG vs. OG, *p* = 0.005) and posttest (HG vs. OG, *p* < 0.001; OWG vs. OG, *p* = 0.001). ^β^ Significantly lower in HG than OG in pre- and posttest (*p* < 0.05). In weight, an interaction effect (group × time) was detected (*p* = 0.028). The Bonferroni test determined significant differences between groups in the pretest and posttest (*p* < 0.05). The OWG (*p* = 0.016) and OG (*p* < 0.001) decreased their weight after the training program. No interaction effect (group × time) was found in other body composition variables (*p* > 0.05).

**Table 3:** Cardiorespiratory and muscular fitness variables.

	Assessment	HG	OWG	OG	P1 ^for interaction^/ES/SP	P2 ^for time/^ES/SP	P3 ^for group^/ES/SP
Participants (n)		10	10	12			
VO_2_peak (mL·kg^−1^·min^−1^)	Pre	30.38 (3.62)	28.66 (2.41)	29.45 (2.89)	0.008	0.005	0.763
	Post	29.87 (2.39)	30.13 (3.09) *	30.93 (2.46) *	0.29/0.83	0.24/0.83	0.02/0.09
Strength DA (repetitions)	Pre	21.00 (2.21)	21.60 (3.17)	18.55 (2.54)	0.696	0.003	0.031
	Post	23.20 (2.04)	22.80 (3.91)	20.91 (2.77)	0.03/0.10	0.27/0.87	0.22/0.66
Strength NDA (repetitions)	Pre	21.20 (1.23)	21.80 (3.43)	18.09 (3.11)	0.754	0.001	0.01
	Post	23.00 (2.11)	23.40 (3.95)	20.64 (2.46)	0.02/0.09	0.32/0.94	0.28/0.81
8 foot UP & Go (seconds)	Pre	5.93 (0.49)	6.00 (0.59)	6.12 (0.94)	0.481	0.008	0.786
	Post	5.34 (0.31)	5.72 (0.62)	5.24 (1.77)	0.05/0.17	0.22/0.79	0.02/0.08
Jump height (cm)	Pre	9.21 (2.05)	9.84 (1.79)	11.05 (4.23)	0.001	0.001	0.226
	Post	10.66 (2.10) *	9.93 (2.10)	11.17 (4.53)	0.17/0.95	0.13/0.92	0.04/0.31
Power output (watts)	Pre ^†^	390.64 (66.35)	466.28 (60.33)	554.76 (173.51)	0.044	0.005	<0.001
	Post	418.25 (65.31) *	462.99 (64.41)	577.03 (168.11) *^‡^	0.08/0.60	0.09/0.82	0.29/0.99

Data are provided as mean ± standard deviation (SD). Abbreviations: DA = dominant arm; ES = effect size; HG = healthy group; NDA = no dominant arm; OG = obesity group; OWG = overweight group; SP = statistical power. P1 = *p*-value for group × time interaction effect; P2 = *p*-value for time effect; P3 = *p*-value for group effect. Bonferroni’s multiple comparisons determined: * Significant differences compared to pretest (*p* < 0.05). ^†^ Significant differences between experimental groups in pretest (*p* < 0.05). ^‡^ Significant differences compared to HG (*p* < 0.001) and OWG (*p* = 0.001) in posttest.

## Data Availability

Not applicable.
